# Оценка краткосрочной и долгосрочной ремиссии акромегалии после эндоскопической трансназальной аденомэктомии

**DOI:** 10.14341/probl13192

**Published:** 2022-12-20

**Authors:** А. С. Луценко, Ж. Е. Белая, Е. Г. Пржиялковская, А. М. Лапшина, А. Г. Никитин, В. Н. Азизян, О. В. Иващенко, А. Ю. Григорьев, Г. А. Мельниченко

**Affiliations:** Национальный медицинский исследовательский центр эндокринологии; Национальный медицинский исследовательский центр эндокринологии; Национальный медицинский исследовательский центр эндокринологии; Национальный медицинский исследовательский центр эндокринологии; Научно-исследовательский институт пульмонологии; Национальный медицинский исследовательский центр эндокринологии; Национальный медицинский исследовательский центр эндокринологии; Национальный медицинский исследовательский центр эндокринологии; Национальный медицинский исследовательский центр эндокринологии

**Keywords:** акромегалия, нейрохирургическое лечение, аденома гипофиза, микроРНК

## Abstract

ОБОСНОВАНИЕ. Нейрохирургическое лечение является наиболее эффективным методом терапии акромегалии. Учитывая преобладающую долю макроаденом у пациентов с акромегалией, оперативное лечение не всегда является успешным, даже при экспертном уровне нейрохирурга. Определение частоты ремиссии акромегалии после хирургического лечения и поиск дооперативных предикторов эффективности являются актуальными задачами современных исследований.ЦЕЛЬ. Оценить краткосрочную и долгосрочную ремиссию акромегалии после эндоскопической трансназальной аденомэктомии в условиях высокоспециализированного стационара и определить предикторы эффективности нейро хирургического вмешательства.МАТЕРИАЛЫ И МЕТОДЫ. Проведено одноцентровое проспективное неконтролируемое исследование. В выборку включались пациенты с активной стадией акромегалии, не получавшие медикаментозную терапию аналогами соматостатина, направленные на эндоскопическую транссфеноидальную аденомэктомию. Экспрессия микроРНК плазмы проведена методом количественной ПЦР с обратной транскрипцией. Послеоперационные образцы аденом направлены на иммуногистохимическое исследование с определением морфологического варианта и экспрессии рецепторов соматостатина 2 и 5 подтипов.РЕЗУЛЬТАТЫ. В исследование включены 44 пациента, из них 32,8% мужчин, медиана возраста составила 47,0 [34,0; 55,0], уровень инсулиноподобного фактора роста 1-го типа (ИФР-1) — 744,75 нг/мл [548,83; 889,85], соматотропного гормона (СТГ) — 9,5 нг/мл [4,94; 17,07], объем опухоли — 832 мм3 [419,25; 2532,38]. Ранняя послеоперационная ремиссия достигнута у 35 больных (79,5%). Пациенты, достигшие кратковременной ремиссии, имели более высокий уровень ИФР-1 и базальный СТГ. Медиана наблюдения составил 19,0 мес [12,5; 29,0]. Длительная ремиссия достигнута у 61,4% (27 больных), ремиссии не было у 9 (20,5%), рецидив выявлен у 2 пациентов (4,5%), 6 потеряны для наблюдения (13,6%). У пациентов с долгосрочной ремиссией отмечались более низкие базальные уровни СТГ и ИФР-1. Различий по уровням исследованных микроРНК не выявлено. Проведена оценка прогностической ценности базального СТГ до операции в определении долгосрочной ремиссии: площадь под кривой 0,811 (95% ДИ 0,649; 0,973). Пороговое значение 15,55 нг/мл соответствовало чувствительности 70,0 (34,8; 93,3)%, специфичности — 85,7 (67,3; 96,0)%, точности — 81,6 (65,7; 92,3)%, прогностической ценности положительного результата — 63,6 (39,3; 82,5)%, прогностической ценности отрицательного результата — 88,9 (75,4; 95,4)%.ЗАКЛЮЧЕНИЕ. Частота краткосрочной и долгосрочной ремиссии после эндоскопической транссфеноидальной аденомэктомии в изученной когорте составила 79,5 и 61,4% соответственно и сравнима с литературными данными нейрохирургических центров экспертного уровня. Базальный СТГ демонстрирует потенциальную ценность в прогнозировании долгосрочной ремиссии акромегалии, однако для получения более точных отрезных значений необходимы дальнейшие исследования на расширенной выборке.

## Abstract (en)

BACKGROUND. Neurosurgery is the most effective treatment for acromegaly. As most of the patients present with macroadenomas, surgical treatment is not always successful, even with the expert level of a neurosurgeon. Assessment of the postoperative remission rates in acromegaly preoperative predictors of treatment efficacy is an urgent task of modern research. AIM: To assess the short-term and long-term remission of acromegaly after endoscopic transnasal adenomectomy in a tertiary medical center and assess preoperative predictors of the treatment effectiveness.MATERIALS AND METHODS: A single-center, prospective, uncontrolled study was conducted. We included patients with active acromegaly who did not receive medical therapy with somatostatin analogues and were referred for endoscopic transsphenoidal adenomectomy. Plasma miRNA expression was assessed by quantitative reverse transcription PCR. Postoperative samples of adenomas were sent for study, with the determination of the immunohistochemical staining for somatostatin receptors 2 and 5 subtypes and morphology was performed on postoperative adenoma samples.RESULTS: The study included 44 patients: 32.8% men, median age 47.0 [34.0; 55.0], IGF-1 744.75 ng/ml [548.83;889.85], growth hormone 9.5 ng/ml [4.94; 17.07]. Tumor volume 832 mm3 [419.25; 2532.38]. Early postoperative remission was achieved in 35 patients (79.5%). Patients who achieved short-term remission had higher IGF-1 and basal growth hormone levels. Median follow-up was 19.0 months [12.5;29.0]. Long-term remission was achieved in 61.4% (27 patients), no remission in 9 (20.5%), recurrency in 2 patients (4.5%), 6 patients were to follow-up (13.6%). In patients with long-term remission, we observed lower growth hormone and IGF-1 levels. No differences in miRNA expression was observesd. The predictive value of basal GH before surgery for long-term remission was assessed: area under the curve 0.811 (95% CI: 0.649; 0.973). A cut-off value of 15.55 ng/mL corresponded to a sensitivity of 70.0% (34.8%; 93.3%), a specificity of 85.7% (67.3%; 96.0%), an accuracy of 81.6% (65 .7%; 92.3%), PPV 63.6% (39.3%; 82.5%), NPV 88.9% (75.4%; 95.4%).CONCLUSION: Rates of short-term and long-term remission after endoscopic transsphenoidal adenomectomy in our cohort is 79,5% и 61,4%, respectively, and is comparable with literature data for expert pituitary centers. Preoperative GH shows potential value in predicting the long-term remission of acromegaly, but further studies in a larger sample are needed to obtain more accurate cut-off values.

## ОБОСНОВАНИЕ

Нейрохирургическое лечение по-прежнему является наиболее эффективным методом терапии акромегалии. В настоящее время основными подходами к хирургическому лечению являются микроскопическая и эндоскопическая трансназальная аденомэктомия (ТНАЭ). Хотя частота ремиссии среди пациентов с микроаденомами достаточно высока и достигает 75%, эффективность хирургического лечения макроаденом, в зависимости от характера роста, значительно меньше — 33–44,5%, что имеет критическое значение в случаях акромегалии, где частота макроаденом преобладает [1]. При этом, даже если оперативное лечение неэффективно, гистологическое и иммуногистохимическое (ИГХ) исследования удаленной опухоли позволяют оценить морфологическое строение и рецепторный профиль и определить оптимальное направление дальнейшего ведения [2].

В настоящее время существуют клинические, радиологические, иммуногистохимические и генетические предикторы эффективности нейрохирургического лечения акромегалии. Клиническими факторами неэффективности являются мужской пол, молодой возраст, большой размер аденомы, высокие предоперационные значения соматотропного гормона (СТГ) и инсулиноподобного фактора роста 1-го типа (ИФР-1), однако данные показатели не соответствуют многим критериям идеального биомаркера. Основным радиологическим предиктором является степень инвазии аденомы в кавернозный синус, наиболее часто оцениваемая по шкале Knosp. Индекс пролиферации Ki-67 является прогностическим маркером эффективности лечения акромегалии, однако оценку данного показателя можно провести только после хирургического вмешательства. Полиморфизмы гена ИФР-1 и рецептора гормона роста относятся к генетическим предикторам эффективности оперативного лечения [3][4].

Chen C.J. и соавт. провели систематический обзор с целью сравнить исходы микрохирургической и эндоскопической ТНАЭ: анализ включал суммарно 52 исследования, 4375 пациентов. Авторы систематического обзора делают вывод, что оба подхода являются применимыми для лечения акромегалии и демонстрируют схожие показатели ремиссии. При этом опыт хирурга и владение техникой оказывают значимое влияние на исходы в обоих случаях [5].

Определение частоты краткосрочной и долгосрочной ремиссии акромегалии после нейрохирургического вмешательства является актуальной задачей, позволяющей сопоставить эффективность радикального лечения между центрами и определить возможные пути улучшения данных показателей. Поиск прогностических маркеров эффективности нейрохирургического лечения акромегалии также направлен на определение пациентов, нуждающихся в более активном наблюдении после операции.

## ЦЕЛЬ ИССЛЕДОВАНИЯ

Оценить краткосрочную и долгосрочную ремиссию акромегалии после эндоскопической ТНАЭ в условиях высокоспециализированного стационара и определить предикторы эффективности нейрохирургического вмешательства.

## МАТЕРИАЛЫ И МЕТОДЫ

## Место и время проведения исследования

Место проведения. Отделение нейроэндокринологии и остеопатий ФГБУ «НМИЦ эндокринологии» Минздрава России.

Время исследования. Период набора: декабрь 2016–ноябрь 2018 гг.

## Изучаемые популяции

В рамках исследования изучалась популяция пациентов с акромегалией, направленных на нейрохирургическое лечение.

Критерии включения: мужской и женский пол, возраст 18 лет и старше, активная стадия акромегалии (код МКБ-10 E22.0), подтвержденная характерными клиническими проявлениями, повышением уровня ИФР-1 (согласно возрастному референсному диапазону) и отсутствием подавления секреции СТГ менее 1,0 нг/мл в ходе перорального глюкозотолерантного теста (ПГТТ).

Критерии исключения: прием аналогов соматостатина в анамнезе или на момент включения в исследование, лучевая терапия в анамнезе, акромегалия вследствие генетических синдромов, период беременности и лактации, психические заболевания, онкологические заболевания, наличие острых заболеваний и декомпенсации сопутствующих заболеваний на момент включения.

Критерии прекращения участия в исследовании: потеря для наблюдения, смерть.

## Способ формирования выборки из изучаемой популяции (или нескольких выборок из нескольких изучаемых популяций)

Выборка формировалась сплошным способом.

## Дизайн исследования

Одноцентровое проспективное одновыборочное неконтролируемое исследование.

## Методы

ИФР-1 (возраст-специфические референсные диапазоны: 18–20 лет — 127–584 нг/мл; 21–25 лет — 116–358 нг/мл; 26–30 лет —117–329 нг/мл; 31–35 лет — 115–307 нг/мл; 36–40 лет — 109–284 нг/мл; 41–45 лет — 101–267 нг/мл; 46–50 лет — 94–252 нг/мл; 51–55 лет — 87–238 нг/мл; 56–60 лет — 81–225 нг/мл и 61–65 лет — 75–212 нг/мл) и СТГ (референсный диапазон: 0,6–6,9 нг/мл) измеряли иммунохемилюминесцентным методом на аппарате Liaison. Измерение уровня пролактина проводилось иммунохемилюминесцентным методом на автоматизированной системе Vitros 3600 (референсный диапазон: 69–340 мЕд/л).

Магнитно-резонансная томография (МРТ) головного мозга проводилась на магнитно-резонансном томографе Magnetom Harmony 1.0Т фирмы Siemens (Германия) с введением гадолиниевого контрастного препарата. Объем опухоли (V) был рассчитан способом, предложенным G. Di-Chiro и K.B. Nelson для измерения объема гипофиза:

V=0,5×LxW×T,

где L — высота аденомы; W — ширина; T — переднезадний размер (толщина); выраженные в мм [6].

Гистологическое и ИГХ-исследование проведены на послеоперационных образцах с наличием опухолей, объем которых был достаточен для гистологического и ИГХ-исследований. Приготовление гистологических препаратов проводилось по стандартной методике с использованием гистопроцессора (Leica asp200) с проведением дегидратации полученных фрагментов ткани опухоли в абсолютном спирте в течение 12 ч, затем ткань заливалась в парафиновые блоки, после чего из парафиновых блоков производились срезы толщиной не более 5 мкм. Далее срезы депарафинизировали, окрашивали гематоксилином и эозином и проводили ИГХ-реакции с антителами к СТГ (разведение 1:400, Dako, поликлональные кроличьи), пролактину (разведение 1:600, Dako, поликлональные кроличьи), низкомолекулярному цитокератину (разведение 1:100, моноклональные мышиные, клон CAM 5.2 Cell Marque), рецепторам соматостатина 2-го подтипа (разведение 1:100, Epitomics, моноклональные кроличьи, клон EP 149), рецепторам соматостатина 5 подтипа (разведение 1:100, abcam, моноклональные кроличьи, клон UMB-5), с демаскирующей обработкой буфером с высоким pH. ИГХ-исследование выполнено с помощью автоматизированного аппарата (иммуностейнер Leica Bond Max) по стандартному протоколу.

В соответствии с морфологической классификацией ВОЗ от 2017 г. [7] в исследованных образцах ткани опухоли гипофиза были разделены на плотногранулированные соматотропиномы (ПГС), редкогранулированные соматотропиномы (РГС), маммосоматотропиномы (МС), смешанные сомато- и лактотрофные аденомы (ССЛ). ПГС представлены оксифильными клетками (при окраске гематоксилином и эозином) с интенсивным окрашиванием цитоплазмы большинства опухолевых клеток сантителами к СТГ и низкомолекулярному цитокератину (при ИГХ), РГС — опухоли гипофиза из оксифильных и хромофобных клеток (при окраске гематоксилином и эозином) с очаговой или слабо выраженной иммуноэкспрессией СТГ цитоплазмы опухолевых клеток и наличием фиброзных телец в более 75% клеток опухоли. МС состоят из одной популяции клеток, способных экспрессировать СТГ и пролактин. ССЛ состоят из двух популяций клеток, каждая из которых имеет позитивное окрашивание на СТГ или пролактин.

В качестве контроля для антител к СТГ, САМ 5.2 и пролактину использовали ткань трупного аденогипофиза.

## Генетические исследования

МикроРНК в данной когорте ранее изучались в двухэтапном исследовании [8][9]. В настоящем исследовании проведена оценка уровней микроРНК у пациентов в зависимости от достижения краткосрочной и долгосрочной ремиссии после нейрохирургического вмешательства.

Выделение микроРНК проводилось из 200 мкл плазмы с использованием наборов miRNeasy Serum/Plasma Kit (Qiagen, Германия), согласно инструкции производителя, на автоматической станции QIAcube (Qiagen, Германия). Для предотвращения деградации в выделенную РНК добавляли 1 ед. RiboLock RNase Inhibitor (Thermo Fisher Scientific, США) на 1 мкл раствора нуклеиновых кислот. Концентрацию суммарной РНК в водном растворе оценивали на спектрофотометре NanoVue Plus (GE Healthcare, Великобритания).

Анализ экспрессии микроРНК производили с помощью полимеразной цепной реакции (ПЦР) в режиме реального времени с обратной транскрипцией (RT-qPCR). Исследование проведено на оборудовании StepOnePlus (Applied Biosystems, США) снаборами TaqMan Advanced miRNA cDNA Synthesis (Thermo Fisher Scientific, США) и TaqMan Advanced miRNA (Thermo Fisher Scientific, США), согласно инструкциям производителей. Общий объем ПЦР составлял 20 мкл. Условия амплификации фрагментов ДНК: 95°C/20 с — 1 цикл; 95°C/1 с, 95°C/20 с — 40 циклов. Данные экспрессии нормализованы с использованием геометрической средней референсной микроРНК (cel-miR-39-3p). Для получения значений пороговых циклов (cycle threshold, Ct) использовано программное обеспечение SDS (версия 2.3, Applied Biosystems, США). Значение Ct<35 выбрано в качестве порогового для определения экспрессии микроРНК. Для сравнительной оценки циркулирующих микроРНК использован метод дельта-Ct, выполненный в пакете ddCT версии 1.30.0.

Количественные данные, полученные из исходного анализа RT-qPCR, были преобразованы с использованием log2 fold changes (FC) в сравнении с контролем (cel-39), чтобы обеспечить прямое сравнение между группами. Затем значения FC2 сравнивались между каждой парой групп с использованием независимого t-критерия выборки с вычислением нескорректированного значения p и скорректированного значения p для исключения ложного обнаружения различий при множественных сравнениях с использованием метода Бенджамини–Хохберга.

Критерии оценки исходов. Критерием ремиссии акромегалии в раннем послеоперационном периоде считалось подавление уровня СТГ в ходе ПГТТ менее 1,0 нг/мл (строгий критерий ремиссии — менее 0,4 нг/мл). Критерий долгосрочной послеоперационной ремиссии — нормализация уровня ИФР-1 согласно возраст-специфическому референсному диапазону.

## Статистический анализ

Основные количественные характеристики пациентов представлены в виде среднего (М) или медианы (Ме) и 95% доверительного интервала (95% ДИ) или межквартильного размаха (25; 75 процентиль — Q25; Q75) в зависимости от характера распределения и выбранного метода анализа. Соотношения качественных признаков представлены в виде долей (%).

Сравнение между количественными параметрами пациентов и группой контроля проводили с использованием непарных двухсторонних t-тестов или критерия Манна–Уитни. Точный критерий Фишера был использован для сравнения двух независимых групп для качественных параметров. Статистически значимым признавался уровень ошибки первого рода менее 5% (p<0,05). Для коррекции проблемы множественных сравнений применялась поправка Бонферрони. После применения поправки значения p в диапазоне между рассчитанным и 0,05 интерпретировались как статистическая тенденция.

Статистический анализ и графический вывод результатов выполнялись с использованием программного обеспечения R версии 3.4.0 (2017-04-21).

Корреляционный анализ проведен с использованием коэффициента ранговой корреляции тау-b Кендалла.

ROC-анализ с расчетом чувствительности, специфичности, прогностической ценности положительного результата (ПЦПР) и прогностической ценности отрицательного результата (ПЦОР) и их 95% доверительного интервала (ДИ) по методу Клоппера–Пирсона проводился для оценки прогностической ценности базального СТГ перед оперативным лечением.

Отрезные значения отбирались согласно критерию Юдена. Затем производилось вычисление основных показателей эффективности диагностического метода: диагностической чувствительности, диагностической специфичности, общей точности, ПЦПР и ПЦОР.

## Этическая экспертиза

Проведение исследования одобрено локальным этическим комитетом ФГБУ «НМИЦ эндокринологии» Минздрава России: «Планируемая научная работа соответствует этическим стандартам добросовестной клинической практики и может быть проведена на базе отделения нейроэндокринологии и остеопатий ФГБУ “НМИЦ эндокринологии” Минздрава России» (протокол заседания №20 от 14 декабря 2016 г.).

Добровольное информированное согласие было подписано всеми пациентами, включенными в исследование.

## РЕЗУЛЬТАТЫ

## Краткосрочная ремиссия после эндоскопической ТНАЭ

В исследование включены 44 пациента, основные характеристики когорты и краткосрочные результаты нейрохирургического лечения представлены в таблице 1. Значения экспрессии исследованных микроРНК представлены в таблице 2.

**Table:** Таблица 1. Основные характеристики пациентов с акромегалией и частота краткосрочной ремиссии после ТНАЭTable 1. Main characteristics of patients with acromegaly and short-term remission rates after endoscopic transnasal adenomectomy

Количество пациентов	44
Пол, м (%):ж (%)	14 (32,8%):30 (68,2%)
Возраст, лет	47,00 [ 34,00; 55,00]
ИМТ, кг/м2	28,25 [ 25,72; 34,50]
ИФР-1, нг/мл	744,75 [ 548,83; 889,85]
СТГ базальный, нг/мл	9,50 [ 4,94; 17,07]
Размер аденомы, n (%): Микроаденома Макроаденома	8 (18,2%) 36 (81,8%)
Объем опухоли (МРТ), мм3	832 [ 419,25; 2532,38]
Ремиссия после нейрохирургического лечения, n (%)	35 (79,5%)
Частота ремиссии в зависимости от размера аденомы, n (%): Микроаденома Макроаденома	7 (87,5%) 28 (77,7%)
Частота развития осложнений после нейрохирургического лечения, n (%): Гипопитуитаризм Несахарный диабет	5 (11,4%) 4 (9,1%)

**Table:** Таблица 2. Экспрессия микроРНК у пациентов с акромегалией, включенных в исследование (n=44)Table 2. MicroRNA expression in patients with acromegaly included in the study (n=44) Примечание. Количественные признаки представлены в виде медианы (Me) и межквартильного диапазона [ Q1; Q3].

МикроРНК	Значение экспрессии
miR-4446-3p	0,274 [ 0,412; 0,561]
miR-215-5p	0,168 [ 0,214; 0,289]
miR-342-5p	0,750 [ 1,139; 1,567]
miR-210-3p	0,992 [ 1,593; 2,163]
miR-146a-5p	0,651 [ 1,116; 1,733]
miR-185-5p	0,967 [ 1,263; 1,461]
miR-34a-5p	0,915 [ 1,132; 1,536]

Далее проведено сравнение пациентов в зависимости от достижения ремиссии в раннем послеоперационном периоде (табл. 3). Пациенты с сохраняющейся активностью заболевания имели статистически значимо более высокий уровень базального СТГ по сравнению с пациентами в ремиссии и более высокий ИФР-1 — на уровне статистической тенденции. Различий по уровням исследованных микроРНК не выявлено.

**Table:** Таблица 3. Сравнительная характеристика пациентов в зависимости от достижения краткосрочной ремиссии акромегалии после нейрохирургического леченияTable 3. Comparative characteristics of patients depending on the achievement of short-term remission of acromegaly after neurosurgical treatment

Параметр	Ремиссия (n=35)	Нет ремиссии (n=9)	p*
Возраст, годы	45 [ 33; 55]	47 [ 29; 53]	0,328
Пол, м (%):ж (%)	10 (28,6%):25 (71,4%)	4 (44,4%):5 (55,6%)	0,434
ИФР-1, нг/мл	737,60 [ 532,10; 876,20]	935,60 [ 649,60; 1186,00]	0,047
Базальный СТГ до операции, нг/мл	8,90 [ 3,74; 15,20]	36,40 [ 9,61; 63,30]	0,001
Макроаденома (%):микроаденома (%)	28 (80%):7 (20%)	8 (89%):1 (11%)	1,000
Объем опухоли	858,00 [ 405,00; 1552,80]	1638,00 [ 360,00; 3450,00]	0,156
ПГС (%):РГС (%)	23 (74,2%):8 (25,8%)	7 (77,8%):2 (22,2%)	1,000
SSTR2, IRS	6,0 [ 4,0; 9,0]	6,0 [ 2,0; 12,0]	0,469
SSTR5, IRS	6,0 [ 2,0; 6,0]	6,0 [ 2,0; 6,0]	0,904
SSTR2/SSTR5	1,3 [ 0,7; 3,0]	1,0 [ 0,5; 3,0]	0,987

При оценке ремиссии с использованием строгого критерия (подавление СТГ в ходе ПГТТ менее 0,4 нг/мл) ранняя послеоперационная ремиссия отмечена у 23 пациентов, отсутствие ремиссии — у 21. Выявлена статистическая тенденция к различиям в уровнях базального СТГ до операции, объеме опухоли и экспрессии SSTR2, а также экспрессии miR-215-5p (табл. 4).

**Table:** Таблица 4. Сравнительная характеристика пациентов после нейрохирургического лечения при использовании строгих критериев ремиссииTable 4. Comparative characteristics of patients after neurosurgical treatment using strict remission criteria

Параметр	Ремиссия (n=23)	Нет ремиссии (n=21)	p*
Возраст, годы	45,0 [ 30,75; 53,0]	45,5 [ 33,5; 55,25]	0,961
Пол, м (%):ж (%)	7 (30,4%):15 (69,6%)	7 (33,3%):14 (66,7%)	1,000
ИФР-1, нг/мл	717,75 [ 500,08; 879,28]	761,1 [ 638,08; 930,88]	0,575
Базальный СТГ до операции, нг/мл	8,99 [ 4,00; 14,25]	22,35 [ 6,57; 43,98]	0,014
Макроаденома (%):микроаденома (%)	17 (73,9%):6 (26,1%)	19 (90,5%):2 (9,5%)	0,240
Объем опухоли	763,5 [ 310,5; 1214,5]	1539,0 [ 763,75; 3775,5]	0,016
ПГС (%): РГС(%)	16 (84,2%):3 (15,8%)	14 (66,7%):7 (33,3%)	0,290
SSTR2, IRS	8,0 [ 6,0; 11,25]	6,0 [ 2,0; 9,0]	0,033
SSTR5, IRS	6,0 [ 2,25; 7,5]	5,5 [ 2,0; 6,0]	0,245
SSTR2/SSTR5	1,5 [ 1,0; 3,75]	1,0 [ 0,5; 3,0]	0,287
miR-215-5p	0,246 [ 1,875; 0,980]	0,199 [ 0,157; 0,236]	0,050

Учитывая значимые различия в уровнях базального СТГ, принято решение изучить прогностическую ценность данного показателя в определении ремиссии акромегалии с использованием ROC-анализа (рис. 1).

**Figure:**
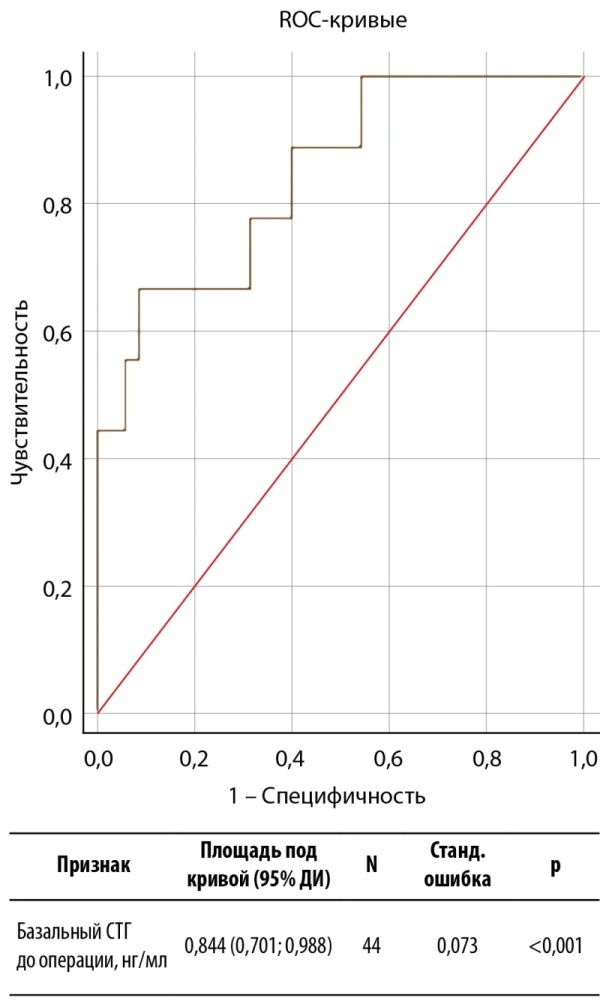
Рисунок 1. ROC-анализ базального СТГ до операции в качестве предиктора ремиссии акромегалии в раннем послеоперационном периоде.Figure 1. ROC analysis of basal GH before surgery as a predictor of remission of acromegaly in the early postoperative period.

Согласно критерию Юдена, выбрано отрезное значение дооперативного уровня СТГ 27,75 нг/мл. Характеристики информативности представлены в таблице 5. ПЦПР полученной модели неудовлетворительная, так как нижняя граница ДИ пересекает 50%. Однако ПЦОР является высокой (91%; 95% ДИ 81–96%), что позволяет рекомендовать модель только для прогнозирования ремиссии с вероятностью от 81% до 96%.

**Table:** Таблица 5. Характеристики прогностической информативности базального СТГ перед операцией в определении ранней послеоперационной ремиссии акромегалииTable 5. Characteristics of the predictive value of basal GH before surgery in determining early postoperative remission of acromegaly Примечание. Представлены характеристики при использовании отрезного значения 27,75 нг/мл. ДЧ — диагностическая чувствительность; ДС — диагностическая специфичность; ОТ — общая точность; ПЦПР — прогностическая ценность положительного результата; ПЦОР — прогностическая ценность отрицательного результата.

	ДЧ (95% ДИ)	ДС (95% ДИ)	ОТ (95% ДИ)	ПЦПР (95% ДИ)	ПЦОР (95% ДИ)
Базальный СТГ до операции	66,7% (29,9–92,5%)	91,4% (76,9–98,2%)	86,4% (72,7–94,8%)	66,7% (38,2–86,6%)	91,4% (80,8–96,4%)

## Долгосрочная ремиссия после эндоскопической ТНАЭ

Медиана периода наблюдения составила 19 мес [ 12,50; 29,00]. При динамическом обследовании у 27 пациентов подтверждена ремиссия заболевания, у двоих выявлен рецидив акромегалии, четверо пациентов были потеряны для наблюдения. Примечательно, что оба случая рецидива заболевания соответствовали строгому критерию ремиссии в раннем послеоперационном периоде. У двоих пациентов отмечено повышение уровня ИФР-1 в течение 3 мес после оперативного вмешательства, при подавлении СТГ в ходе ПГТТ в раннем послеоперационном периоде состояние расценено как дискордантность лабораторных результатов (табл. 6).

**Table:** Таблица 6. Отдаленные результаты эндоскопической ТНАЭ у пациентов с акромегалиейTable 6. Long-term results of endoscopic transnasal adenomectomy in patients with acromegaly Примечание. Количественные признаки представлены в виде медианы (Me) и межквартильного диапазона [ Q1; Q3]. Качественные признаки представлены в виде долей. Для периода наблюдения в круглых скобках приведены минимальные и максимальные значения.*Включены 2 пациента с дискордантностью лабораторных результатов.

Период наблюдения, мес	19,00 [ 12,50; 29,00] (мин. 6; макс. 47)
Отдаленный исход нейрохирургического лечения, n (%):	
Ремиссия	27 (61,4%)
Нет ремиссии*	9 (20,5%)
Рецидив	2 (4,5%)
Потеряны для наблюдения	6 (13,6%)

Из девяти пациентов, которым была назначена медикаментозная терапия аналогами соматостатина пролонгированного действия после оперативного вмешательства, четыре пациента не смогли получить препарат. У трех пациентов, получавших аналоги соматостатина, медикаментозной ремиссии не отмечено. Двое пациентов были потеряны для наблюдения.

Из семи пациентов с сохраняющейся активностью заболевания одному рекомендовано повторное хирургическое вмешательство, одному — лечение аналогами соматостатина, двум — комбинированная терапия аналогами соматостатина иагонистами дофаминовых рецепторов и один пациент направлен на лучевую терапию.

В двух случаях рецидива заболевания пациентам рекомендована медикаментозная терапия аналогами соматостатина. В обоих случаях дискордантности лабораторных показателей состояние расценено как отсутствие ремиссии, рекомендована комбинация аналогов соматостатина и агонистов дофаминовых рецепторов в одном случае и монотерапия агонистами дофаминовых рецепторов в одном случае.

Из пяти пациентов с гипопитуитаризмом (без несахарного диабета) в исходе оперативного лечения в одном случае осложнение носило транзиторный характер. За время наблюдения у всех пациентов с послеоперационным несахарным диабетом отмечался регресс данного осложнения.

Таким образом, из 44 прооперированных пациентов ТНАЭ позволила достичь долгосрочной ремиссии в 27 случаях (61,4%). Сравнительная характеристика пациентов в зависимости от достижения долгосрочной ремиссии представлена в таблице 7.У пациентов без ремиссии значения базального СТГ и ИФР-1 до операции были выше — на уровне статистической тенденции. Различий по уровням исследованных микроРНК также не выявлено.

**Table:** Таблица 7. Сравнительная характеристика пациентов в зависимости от достижения долгосрочной ремиссии акромегалииTable 7. Comparative characteristics of patients depending on the achievement of long-term remission of acromegaly

Параметр	Долгосрочная ремиссия (n=27)	Нет ремиссии (n=11)*	p**
Возраст, годы	45,0 [ 33,0; 55,0]	47,0 [ 29,0; 53,0]	0,874
Пол, м(%):ж(%)	7 (25,9%):20 (74,1%)	5 (45,5%):5 (54,5%)	0,235
ИФР-1 до операции, нг/мл	674,80 [ 482,5; 876,2]	771,0 [ 649,6; 992,0]	0,030
СТГ до операции, нг/мл	8,9 [ 3,76; 11,9]	28,0 [ 6,75; 47,2]	0,006
Макроаденома (%):микроаденома (%)	22 (77,7%):6 (22,3%)	9 (81,8%):2 (18,2%)	1,000
Объем опухоли	858,0 [ 405,0; 1260,0]	1552,8 [ 360,0; 3213,0]	0,339
ПГС (%):РГС (%)	17 (68,0%):8 (32,0%)	8 (80,0%):2 (20,0%)	0,686
SSTR2, IRS	6,0 [ 4,0; 12,0]	6,0 [ 4,0; 8,0]	0,565
SSTR5, IRS	5,0 [ 2,0; 6,0]	6,0 [ 2,0; 6,0]	0,588
SSTR2/SSTR5	1,5 [ 0,67; 3,0]	1,0 [ 0,67; 3,0]	0,637

Учитывая выраженную тенденцию к различиям по уровням базального СТГ до операции, принято решение исследовать предиктивные возможности данного показателя в определении долгосрочной ремиссии акромегалии. ROC-анализ представлен на рисунке 2. Согласно критерию Юдена, оптимальное отрезное значение составило 15,55 нг/мл. Характеристики информативности представлены в таблице 8. Как и в случае модели для определения краткосрочной ремиссии, ПЦПР данной модели неудовлетворительная, так как нижняя граница ДИ пересекает 50%. При этом ПЦОР является высокой (88%, 95% ДИ 75–95%), что также позволяет рассматривать модель только для прогнозирования ремиссии, то есть исключения отсутствия ремиссиис вероятностью от 75 до 95%.

**Figure:**
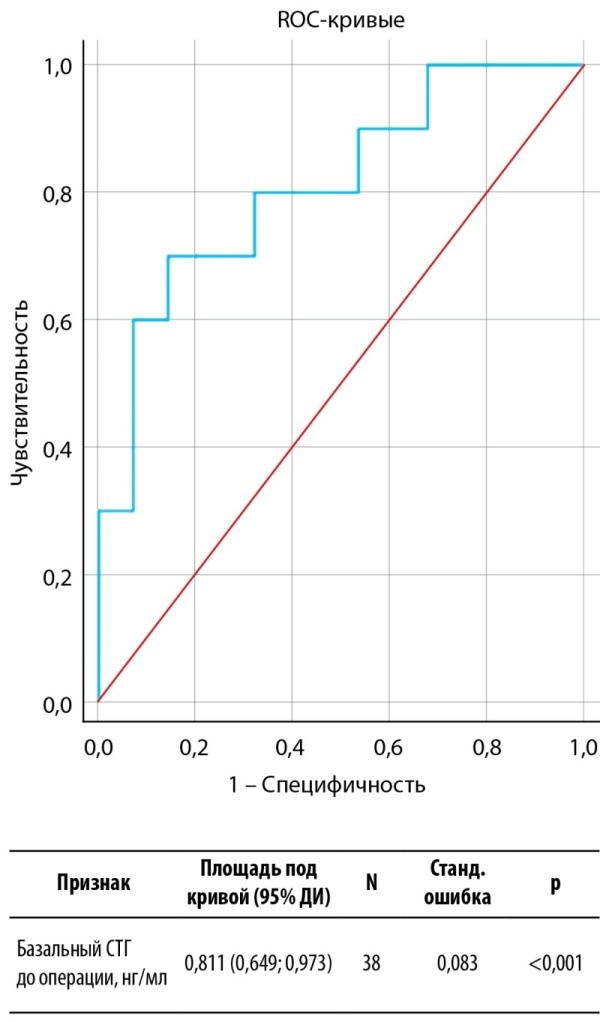
Рисунок 2. ROC-анализ базального СТГ до операции в качестве потенциального предиктора долгосрочной ремиссии акромегалии.Figure 2. ROC analysis of basal GH before surgery as a potential predictor of long-term remission of acromegaly.

**Table:** Таблица 8. Характеристики прогностической информативности базального СТГ перед операцией в определении долгосрочной послеоперационной ремиссии акромегалииTable 8. Characteristics of the prognostic value of basal GH before surgery in determining the long-term postoperative remission of acromegaly Примечание. Представлены характеристики при использовании отрезного значения 15,55 нг/мл. ДЧ — диагностическая чувствительность; ДС — диагностическая специфичность; ОТ — общая точность; ПЦПР — прогностическая ценность положительного результата; ПЦОР — прогностическая ценность отрицательного результата.

	ДЧ (95% ДИ)	ДС (95% ДИ)	ОТ (95% ДИ)	ПЦПР (95% ДИ)	ПЦОР (95% ДИ)
Базальный СТГ до операции	70,0% (34,8–93,3%)	85,7% (67,3–96,0%)	81,6% (65,7–92,3%)	63,6% (39,3–82,5%)	88,9% (75,4–95,4%)

## ОБСУЖДЕНИЕ

## Репрезентативность выборок

По данным исследования на популяции из 3173 пациентов с акромегалией из 10 стран [10], медиана возраста пациентов на момент постановки диагноза составила 45,2 года, в 71,8% случаев на момент постановки диагноза выявлены макроаденомы, что согласуется с возрастом пациентов и данными МРТ в нашей когорте.

## Сопоставление с другими публикациями

Согласно систематическому обзору исходов и осложнений эндоскопической ТНАЭ по поводу акромегалии, общая частота ранней и долгосрочной ремиссии составляет 57,4 и 70,2% соответственно. Для макроаденом данные показатели составляют 40,2 и 61,5%, для микроаденом — 76,9 и 73,5% [5]. Нами получены более высокий процент краткосрочной ремиссии и сопоставимые показатели по долгосрочной ремиссии. Частота гипопитуитаризма и транзиторного несахарного диабета в изученной когорте была несколько выше, чем по данным систематического обзора. Данное явление может объясняться более радикальной операционной тактикой в представленной когорте, что и приводит к более высокой частоте как ремиссии, так и вышеуказанных осложнений. Кроме того, краткосрочная ремиссия оценивалась сразу после операции (на 4–7-й день) по подавлению СТГ в ходе ПГТТ, что не всегда проводится в зарубежных клиниках.

Различия в дооперационных уровнях СТГ и ИФР-1 между пациентами с различной эффективностью нейрохирургического лечения в нашем исследовании согласуются с мировыми данными [11][12]. При этом, в отличие от представленных исследований, мы не обнаружили различий в объеме опухоли между группами и предиктивных возможностей данного показателя. Это может объясняться различными методиками расчета данного показателя и небольшим размером выборки.

В исследовании J.A. Jane, Jr., и соавт. проанализированы исходы эндоскопической ТНАЭ среди 62 пациентов с акромегалией с использованием строгого критерия ремиссии (подавление СТГ в ходе ПГТТ менее 0,4 нг/мл). Между пациентами времиссии после нейрохирургического вмешательства и пациентами, у которых ТНАЭ была неэффективна, так же, как и в нашем исследовании, отмечались различия в дооперационных уровнях СТГ и ИФР-1, а также в объеме опухоли. Кроме того, дооперационные уровни СТГ и ИФР-1 являлись предикторами ремиссии заболевания [12] .

В исследовании Shun Yao и соавт. проводился ретроспективный анализ 546 случаев акромегалии с целью поиска независимых предикторов ремиссии акромегалии. Значение дооперационного уровня СТГ более 28 нг/мл являлось предиктором отсутствия ремиссии после нейрохирургического лечения, что согласуется с полученной нами отрезной точкой данного показателя для определения краткосрочной ремиссии [13].

## Клиническая значимость результатов

Результаты нашего исследования представляют частоту краткосрочной и долгосрочной ремиссии акромегалии после эндоскопической ТНАЭ в высокоспециализированном учреждении и сопоставимость с общемировыми данными.

Определение отрезного значения базального СТГ для определения долгосрочной ремиссии может иметь клиническое значение после валидации результатов на более широкой выборке пациентов и в дальнейшем позволит определять пациентов более высокого риска, требующих активного наблюдения.

## Ограничения исследования

Исследование ограничено небольшим объемом выборки и наличием в выборке пациентов, потерянных для наблюдения. Однако доля данных пациентов сопоставима с данными литературы [14]. Для дальнейшего определения оптимального отрезного значения базального СТГ в качестве предиктора долгосрочной ремиссии необходима расширенная выборка пациентов, однако наши результаты позволяют наметить отправную точку для дальнейших работ.

## ЗАКЛЮЧЕНИЕ

По данным проведенного исследования, частота ремиссии акромегалии после эндоскопической ТНАЭ составила 79,5% при оценке в раннем послеоперационном периоде и 61,4% в ходе долгосрочного наблюдения, что соответствует мировым показателям центров экспертного уровня.

Использование отрезного значения базального СТГ до операции 27,75 нг/мл позволяет предсказывать наличие или отсутствие долгосрочной ремиссии с общей точностью 81,6% (65,7–92,3%), ПЦПР 63,6% (39,3–82,5%) и ПЦОР 88,9% (75,4–95,4%), однако требует валидации на расширенной выборке.

## ДОПОЛНИТЕЛЬНАЯ ИНФОРМАЦИЯ

Источники финансирования. Данная работа выполнена при финансовой поддержке Российского научного фонда, грант №19-15-00398.

Конфликт интересов. Авторы декларируют отсутствие явных и потенциальных конфликтов интересов, связанных с содержанием настоящей статьи.

Участие авторов. Все авторы одобрили финальную версию статьи перед публикацией, выразили согласие нести ответственность за все аспекты работы, подразумевающую надлежащее изучение и решение вопросов, связанных с точностью или добросовестностью любой части работы.
